# 

**Published:** 1901-07-13

**Authors:** 


### NOTICE TO CORRESPONDENTS.

**All MSS., Letters, Books for Review, and other matters intended for the Editor should be addressed THE EDITOR, "THE HOSPITAL," THE HOSPITAL BUILDING, 28 & 29 SOUTHAMPTON STREET, STRAND, W.C.**

**The Editor cannot undertake to return rejected MS., even when accompanied by stamped directed envelope.**

**Notice to Advertisers and Subscribers.**

All Advertisements, Orders for Copies of THE HOSPITAL, and Business Communications should be addressed to **THE MANAGER (Not to the Editor)**, THE HOSPITAL, 28 & 29 Southampton Street, Strand, London, W.C.

The Cost of Subscription, post free, is as follows:—Per week, 2½d.; per quarter, 2s. 8d.; per half-year, 5s. 3d.; per annum, 10s. 6d. Foreign:—Per annum, 16s. 2d., payable, in all cases, in advance.

**NOTICE.—Vol. XXIX. of "The Hospital" (October 1900, to March, 1901), in handsome cloth binding is now on sale at the Office of this Journal, price, 7s. 6d. Cases for binding the Numbers contained in this Volume 1s. 6d. Index to Vol. XXIX., 2½d., post free.**

**The Monthly Part for July is now ready. Price 6d., by post 8d.**

### Scraps and Gleanings.

Messrs. Maple and Co., Limited, of Tottenham Court Road, London and Paris, have been appointed upholsterers to His Majesty, King Edward VII.

Her Majesty Queen Alexandra has graciously accepted a copy of the *Edition de luxe* of "The May Book," compiled by Mrs. Aria in aid of the Charing Cross Hospital.

The Committee of Queen Charlotte's Hospital have received the sum of £775, being the gross proceeds of the tableau vivants arranged by Mrs. Stephen Schilizzi, and given at the Savoy Hotel last week on behalf of the Hospital.

At a recent special meeting of the Management Committee of the Swansea Hospital, held for the purpose of discussing a suggested alteration of the existing laws of the hospital whereby the representation of working men on the Board should be extended, it was decided (1) that the Trades Council should be represented on the Board as members of the Trades Council: (2) that representation must go by contribution; (3) that it was desirable the laws should be altered to give working men a larger representation; and (4) that subscribers of ten guineas should be empowered to nominate a governor.

